# Gait Planning and Stability Control of a Quadruped Robot

**DOI:** 10.1155/2016/9853070

**Published:** 2016-04-10

**Authors:** Junmin Li, Jinge Wang, Simon X. Yang, Kedong Zhou, Huijuan Tang

**Affiliations:** ^1^School of Mechanical Engineering, Xihua University, No. 999 Jinzhou Road, Jinniu District, Chengdu, Sichuan, China; ^2^Advanced Robotics and Intelligent Systems Laboratory, School of Engineering, University of Guelph, Guelph, ON, Canada N1G 2W1

## Abstract

In order to realize smooth gait planning and stability control of a quadruped robot, a new controller algorithm based on CPG-ZMP (central pattern generator-zero moment point) is put forward in this paper. To generate smooth gait and shorten the adjusting time of the model oscillation system, a new CPG model controller and its gait switching strategy based on Wilson-Cowan model are presented in the paper. The control signals of knee-hip joints are obtained by the improved multi-DOF reduced order control theory. To realize stability control, the adaptive speed adjustment and gait switch are completed by the real-time computing of ZMP. Experiment results show that the quadruped robot's gaits are efficiently generated and the gait switch is smooth in the CPG control algorithm. Meanwhile, the stability of robot's movement is improved greatly with the CPG-ZMP algorithm. The algorithm in this paper has good practicability, which lays a foundation for the production of the robot prototype.

## 1. Introduction

The coordinated movement control of multilegged robot has been a difficult problem [1, 2] in the field of robot because a robot needs to make a quick response to the change of external environment and various stimulus. The control strategy based on biological induction is a new control idea that has been gradually carried out in the multilegged robot researches [3–12], in which the alternate rhythmic movement of each leg of the quadruped robot is the most common. Biological studies show that rhythmic movement is usually achieved by CPG (central pattern generator) and can be applied to four-legged robot motion control [13, 14].

The issues concerning the architecture of a CPG model are the choice of type and number of oscillators to use. Matsuoka [15] first achieved CPG output by the use of oscillator model regulation. And this oscillator can only provide a positive output signal, which often makes it difficult to meet the needs of engineering control object. On the basis of Matsuoka's CPG oscillator, Kimura adopted two neurons: flexor and extensor muscles to simulate the movement of the nervous system of animals and to achieve quadruped robot gait control [16]. In addition, he improved the functions of the oscillators by introducing plenty of additional reflex feedback to the controller and performed successful walking tests of Tekken in several terrains. Similar works using these oscillators were introduced by Bailey [10] for controlling insect locomotion and also by Liu et al. [17] for controlling AIBO robot. Huang et al. built mutual suppression oscillator model and control networks of quadruped robot joints based on Matsuoka's neuron and obtained the CPG network parameters of quadruped gait control [2]. But only a single CPG was considered to realize single joint control, which lacks systematic network behavior and is cumbersome to parameter adjustment.

The Hopf oscillator is also popular for modeling the CPG to control legged robots. CPG unit model based on Hopf oscillator was constructed by Santos and Matos [13], which realizes the controllable conditioning of hip drive signals and gait switch of quadruped robot, but it has no effective CPG regulatory networks to control the movement of the other joints directly or indirectly. However, when four Hopf oscillators are connected to obtain phase entrainments, the waveforms of the outputs are changed accordingly. The deformation in the waveforms depends on the connection structure (or the gait) and the connecting weights. Explicit examples can be found elsewhere [18]. In different approaches, instead of generating periodic outputs directly from the dynamic oscillators (Matsuoka and Hopf oscillators), other researchers focused on producing stable phase entrainments using phase oscillators. Tsujita et al. [19] introduced an example of phase oscillators for controlling a quadruped robot. Aoi et al. introduced similar oscillators to control a quadruped robot [20] to perform several locomotion tasks, including dynamic walking and gait transitions. Maufroy et al. [21] used phase modulations to control the posture and rhythmic motions of a quadruped robot.

The Wilson-Cowan neural oscillator is also popular for modeling the CPG to control legged robots. Li et al. presented the Wilson-Cowan neural oscillator controller for quadruped robot rhythmic locomotion control, which is known as a weakly neural network that generates rhythmic movements in locomotion of animals [22]. The harmony motion of one leg from the others is controlled with four Wilson-Cowan neural oscillators. The period and amplitude of the CPG model are easy to control for generating various gaits, but the real time of adjusting the model oscillation system and stability control need to further improve.

In stability control methods, Liu and Chen proposed stability control method of gait based on ZMP (zero moment point) theory to control a quadruped robot, which achieves some success in stability control but lacks efficiency and flexibility of gait planning and gait switch [23].

In this work, a new controller algorithm based on CPG-ZMP (central pattern generator-zero moment point) is put forward in order to realize smooth gait planning and stability control at the same time. At first, a new CPG model controller and its gait switching strategy based on Wilson-Cowan model are presented in order to generate smooth gait and shorten the adjusting time of the model oscillation system. The control signals of knee-hip joints are obtained by the improved multi-DOF reduced order control theory. And, then, adaptive speed adjustment and gait switch are completed by real-time computing of ZMP to realize stability control. Simulation results show that quadruped robot's gait planning is efficiently generated and the gait switch is smooth in the CPG control algorithm. Meanwhile, the stability of the robot movement is improved greatly with CPG-ZMP algorithm. The algorithm has been applied on joint quadruped robot, which greatly improves the stability of movement and the flexibility of gait generation and switch.

## 2. Improved Central Nervous Oscillators Model

**Figure 1:**
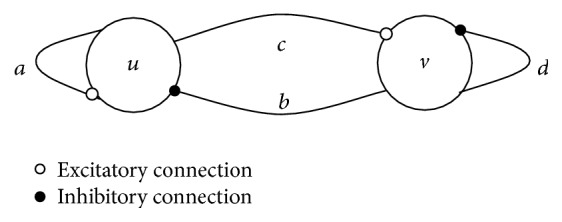
Wilson-Cowan central nervous oscillator model.

The oscillator model presented by Wilson and Cowan is shown in Figure 1, which is composed of excitatory neuron *u* and inhibitory neuron *v*. A stable limit cycle shock is formed by the intercoupling of *u* and *v*.

The model can be described by the following differential equation [22]:

(1) Tududt+u=fμau−bv+Su,Tudvdt+v=fμcu−dv+Sv,fμx=tanh⁡μx,

where *a* is the excitatory connection strength of neuron and *d* is the inhibitory connection strength of neuron, *b* is the inhibitory connection strength of *v* to *u*, *c* is the excitatory connection strength of *u* to *v*, *S*_*u*_ and *S*_*v*_ are the external signals and the *DC* inputs usually, *T*_*u*_ is the rise-time constant of step input, *T*_*v*_ is the fatigue time constant, *f*_*μ*_(*x*) is the coupling function, and *μ* is the gain of *f*_*μ*_(*x*).

In order to apply Wilson-Cowan nervous oscillator on the gait control of quadruped robot, the model is improved as follows:

(1) *p* is introduced as amplitude limiting coefficient to adjust the outputs of *u* and *v*; *y*_out_ is an output of linear synthesis to control the movement of corresponding leg.
(2) Every leg is controlled by a CPG oscillator which is built by weighted directed graph with graph theory.
(3) External feedback of CPG control network is introduced, where *s*_*ik*_ is the reflection information, *g*_*k*_ is coefficient of *s*_*ik*_, and *u* and *v* are the adverse vectors of refection coefficients.

Then the improved oscillator model is described as follows:

(2) Tuiduidt+ui=fμaui−bvi+∑j=1nwijμj+∑k=1msikgk+Sui,Tvidvidt+vi=fμcui−dvi+∑j=1nwijvj−∑k=1msikgk+Svi,fμx=tanh⁡μx,youti=pui−vii,j=1,2,3,4;  k=1,2,3,…,m,

where *i* and *j* are the numbers of central neural oscillators, *w*_*ij*_ is the connection weight between oscillators, and *W* ∈ *R*4 × 4 is a matrix composed of *w*_*ij*_.

**Figure 2:**
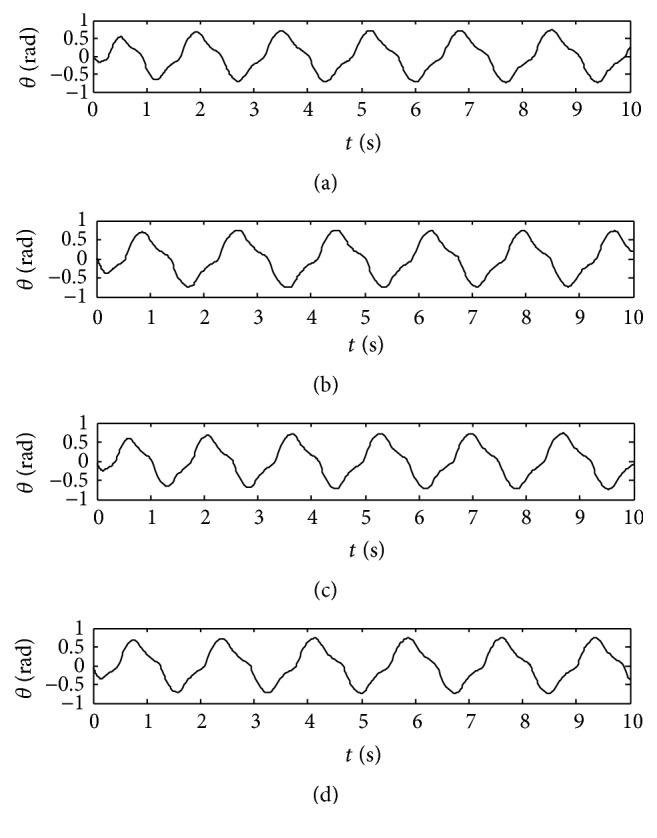
Output curves of walk gait. (a) Left front leg. (b) Right front leg. (c) Right hind leg. (d) Left hind leg.

Output curves of walk gait with the improved central nervous oscillator model are shown in Figure 2. The adjusting time of the improved model is less than 0.5 T, but that of the traditional Wilson-Cowan model is about 1.5 T [22], so the adjusting time of the improved model is shortened greatly.

## 3. Typical Gaits Planning and Transition of Quadruped Robot

Two typical gaits are discussed in this paper, including walk and trot. Walk belongs to a still gait, and each leg puts up and down in turn; the phase difference between legs is a quarter cycle. Trot means that the two diagonal legs put up and down at the same time and is better at energy consumption and belongs to dynamic gait, of which the phase difference between legs is half of cycle.

### 3.1. Typical Gaits Planning

The connection weight matrixes *W*_walk_ and *W*_trot_ are described as follows:

(3) Wwalk=0−0.1−0.1−0.1−0.10−0.1−0.1−0.1−0.10−0.1−0.1−0.1−0.10,Wtrot=0−0.1+0.1−0.1−0.10−0.1+0.1+0.1−0.10−0.1−0.1+0.1−0.10.

**Figure 3:**
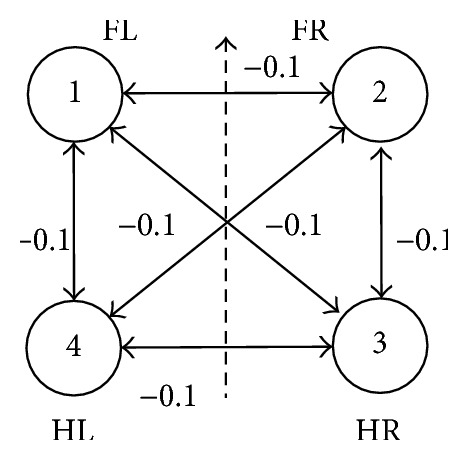
Walk network connection topology structure.

**Figure 4:**
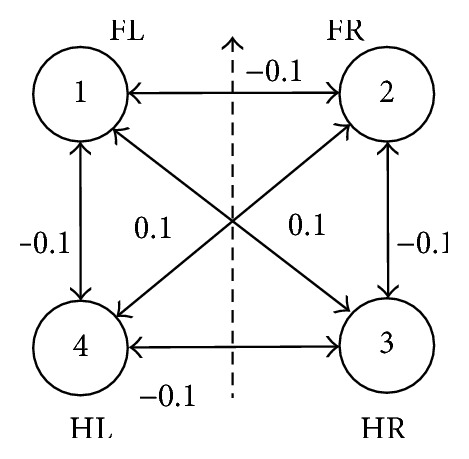
Trot network connection topology structure.

The relative displacement between two legs is a quarter of walk cycle during walking, and each connection among oscillators is inhibitory connection in a full symmetric CPG network. But excitatory connections are adopted in trot. Thus, let the excitatory connection value be +0.1, which is a smaller positive value in *W*, and let the inhibitory connection value be −0.1, which is a smaller negative value. The network topological structure is shown in Figures 3 and 4, respectively.

CPG equation is solved by using the four-order Runge-Kutta. The initial values of matrix are random numbers which are one order of magnitude larger than *S*_*u*_ and *S*_*v*_:

(4) $$\begin{array}{c} \mu_{0}=\left[\begin{array}{cccc} \mu_{1} & \mu_{2} & \mu_{3} & \mu_{4}\\ v_{1} & v_{2} & v_{3} & v_{4} \end{array}\right]=\left[\begin{array}{cccc} 0.1 & 0.25 & 0.1 & 0.18\\ 0.1 & 0.21 & 0.2 & 0.27 \end{array}\right]. \end{array}$$

The parameters in (2) are set in Table 1.

### 3.2. Typical Gaits Transition

**Figure 5:**
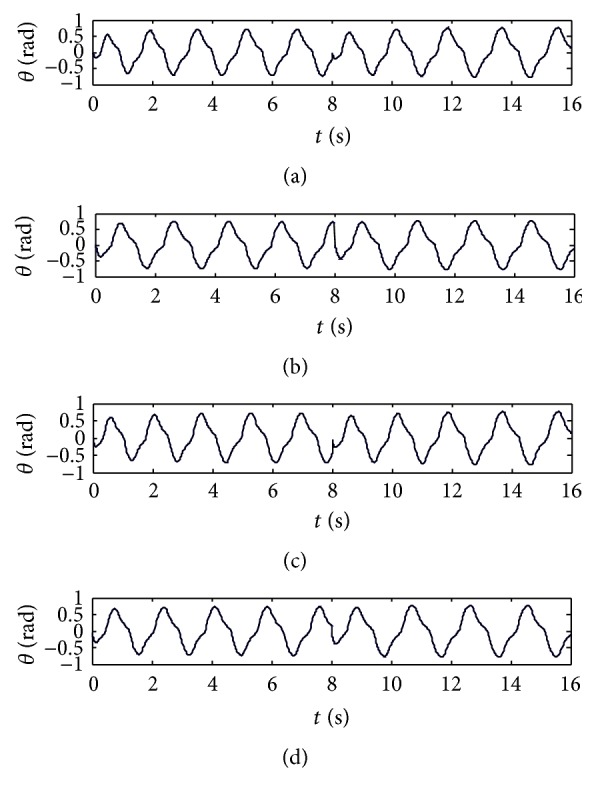
Gait transition from walk to trot. (a) Left front leg. (b) Right front leg. (c) Right hind leg. (d) Left hind leg.

Because there are direct correspondences between the connection weight matrix and gait, we can realize gaits transition by replacing the connection weight matrix. Gait transition curves from walk to trot are shown in Figure 5. Gait transition begins at the time *t* = 8 s. Transition process takes about 0.5 T, but that of the traditional Wilson-Cowan is about 1.5 T [22]. Because the adjusting time of the improved central nervous oscillator model is short, gaits transition is rapid and smooth.

## 4. Multi-DOF Lower-Order Control Method of Quadruped Robot

**Figure 6:**
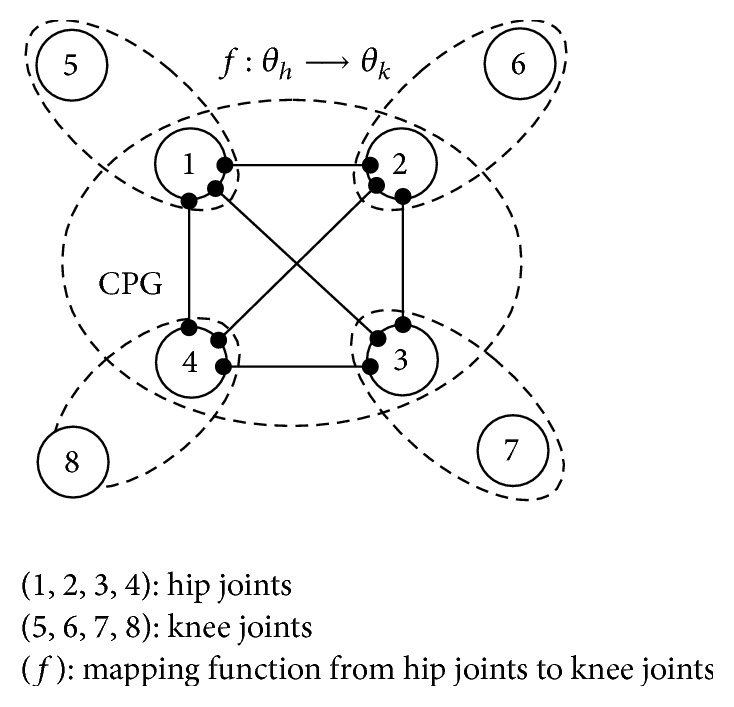
Lower-order control model of quadruped robot.

Lower-order control method of joints in eight DOFs quadruped robot is shown in Figure 6, which builds intercoupling mapping relations between hip joint and knee joint. The control signals of CPG output are used to control the four corresponding hips directly and control the four knee joints indirectly by coupling relationship. CPG oscillation control system couples with hip joints, and hip joints couple with corresponding knee joints. This makes up the intercoupling control system.

### 4.1. Construction of Motion Mapping Functions

The mapping function of knee joint is defined as formula (5), which indicates that the knee joint has movement in swing phase and has only tiny passive movement in support phase as a half-wave function. The tiny movement of knee joint in support phase is ignored in order to simplify control algorithm:

(5) θkt=0θh˙<0sgn⁡φαkAk1−θhtαhAh2θh˙≥0,φ=1,elbow  joint−1,knee  joint,

where *θ*_*h*_, *A*_*h*_, and *α*_*h*_ represent the signals of hip joint and its corresponding amplitude and correction factor, respectively, *θ*_*k*_, *A*_*k*_, and *α*_*k*_ represent the signals of knee joint and its corresponding amplitude and correction factor, respectively, and sgn⁡ (*φ*) is the multiplier of joint configuration form. *θ*_*h*_ is obtained by the Wilson-Cowan neural oscillators. *α*_*k*_ and *α*_*h*_ are positive correlation relationship to *θ*_*k*(*t*)_ and are used to keep the knee joints from touching the ground and obtain mapping signals from the hip joints.

### 4.2. Motion Trajectory Planning of Single Leg

**Figure 7:**
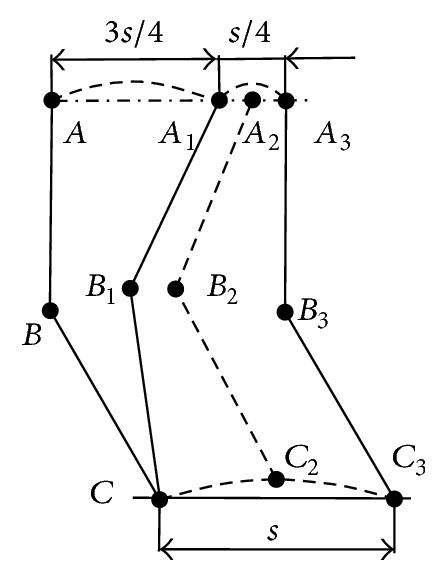
Single leg motion graphic of walk gait.

**Figure 8:**
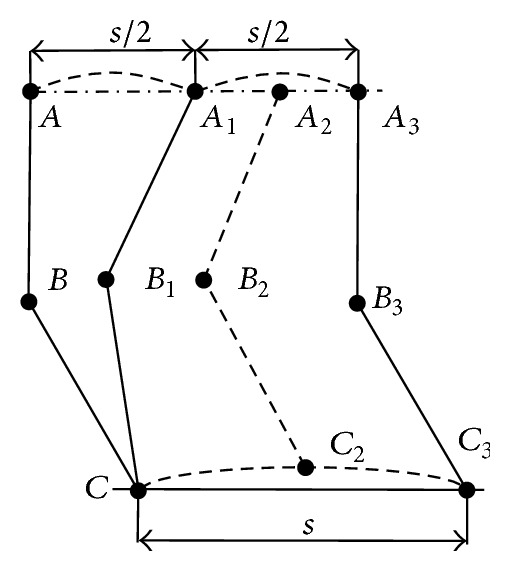
Single leg motion graphic of trot gait.

The duty ratio of walk gait is 3/4, and the motion order of four legs is 1-3-2-4, which realizes reciprocating motion of four legs. The support phase of every leg costs three quarters of the cycle time, and swing phase costs a quarter of the cycle time. The duty ratio of trot gait is 0.5, which means the time of support phase and the time of swing phase are the same. The other two legs sway when the diagonal two legs step on the ground. The whole processes from the beginning of support phase to the end of swing phase for walk gait and trot gait are shown in Figures 7 and 8, respectively.

### 4.3. Motion Parameter Determination of Single Leg

In the virtual prototype of quadruped robot in this paper, the lengths of thigh and shank are *l* = 0.14 m, the speed of walk gait is *v* = 0.12 ms^−1^, and the motion cycle is *T* = 1.5 s.

It can be known by analyzing one leg's motion track that the hip joint's rotor angle is always increasing from support phase's beginning to the end and then is decreasing when the leg is in swing phase. The knee joint has tiny passive rotation range in the support phase, so the knee joint's rotor angle becomes the biggest when the support phase ends and then gets into the swing phase immediately. The knee joint's rotor angle becomes the smallest when the height above the earth for sway leg is in swing phase's midpoint *C*_2_.

As a matter of experience, let the height of leg raise *h* = 0.01 m. Formula (6) can be obtained on the basis of Figure 7:

(6) s=v×T,Ak=∠A1B1C−∠A2B2C2,Ah=π2−∠AA1B1.

Hip joint's swing amplitude *A*_*h*_ is 15.50° and knee joint's swing amplitude *A*_*k*_ is 11.12° by trigonometric function relationships.

When the robot is in trot, let speed *v* = 0.24 ms^−1^ and let motion cycle *T* = 1.2 s, and let the highest height of leg raise above the earth *h* = 0.05 m. Hip joint's swing amplitude *A*_*h*_ is 45° and knee joint's swing amplitude *A*_*k*_ is 14.2° as the same theory.

### 4.4. Motion Track Simulation of Joint

**Figure 9:**
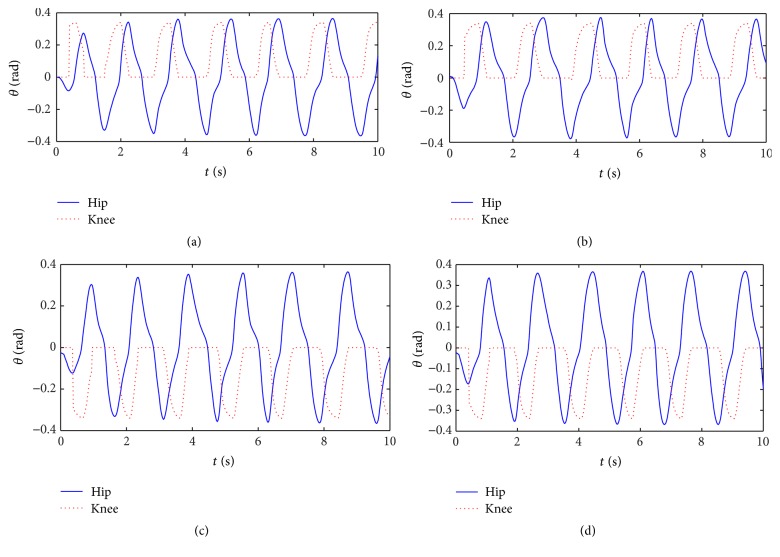
Movement curves of hip-knee joints for walk gait. (a) Left front leg. (b) Right front leg. (c) Right hind leg. (d) Left hind leg.

The configuration form of the robot joint is an inward knee-elbow form. The walk gait matrix *W* is *W*_walk_. The motion tracks of hip joints are controlled by CPG and knee joints are controlled by half-wave functions. The movement curves of hip-knee joints are shown in Figure 9 by MATLAB simulation.

**Figure 10:**
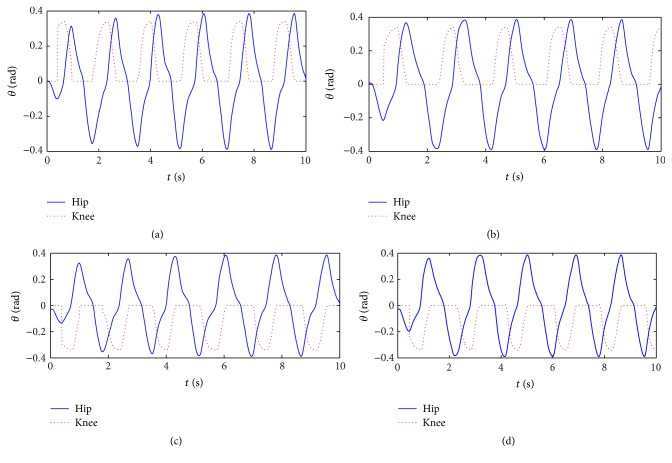
Movement curves of hip-knee joints for trot gait. (a) Left front leg. (b) Right front leg. (c) Right hind leg. (d) Left hind leg.

The zero lines of motion curves in Figure 9 are the balance states of joints. Swing amplitude of hip joints near the balance state is *A*_*h*_, while unilateral swing amplitude of knee joints is *A*_*k*_. The motion curves of front legs' knee joints are in the positive axis and those of the hind legs are in the negative axis. The movement curves of hip-knee joints for trot gait are shown in Figure 10 when gait matrix *W* is *W*_trot_.

## 5. ZMP Model of Quadruped Robot

**Figure 11:**
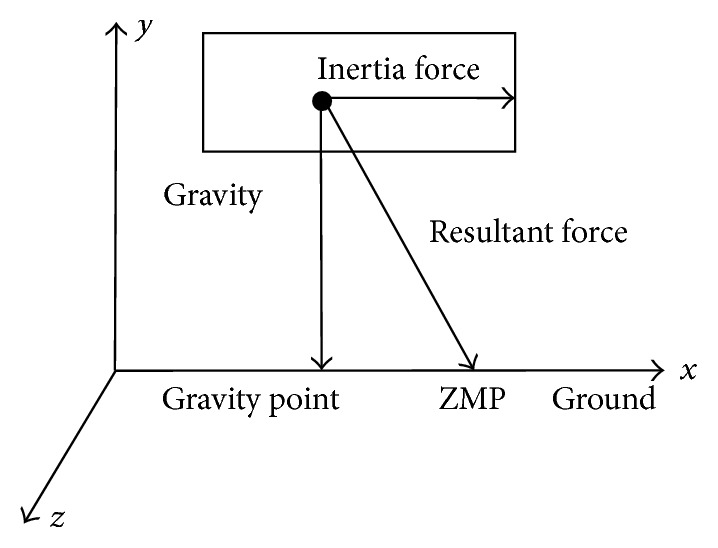
The schematic diagram of ZMP.

Over the past 35 years, there have been many theoretical and experimental studies on the ZMP. To summarize, the ZMP criterion states that if the ZMP is within the support polygon made between the foot and the ground, then stable dynamic walking is guaranteed [23]. The schematic diagram of ZMP is shown in Figure 11.

Assume that the barycentre of robot is in its geometric center and the ground is plane, so the height coordinate *y*_*g*_ of robot centroid above the earth is a constant, and coordinates of ZMP can be obtained by the following formula:

(7) xZMP=xg−x¨ggy·yg,ZZMP=Zg−Z¨ggy·yg,

where *x*_*g*_ is robot barycentre coordinate of *x*-axis, *y*_*g*_ is robot barycentre coordinate of *y*-axis, *z*_*g*_ is robot barycentre coordinate of *z*-axis, ${\ddot{x}}_{g}$> is the acceleration of *x*-axis, and ${\ddot{z}}_{g}$> is the acceleration of *y*-axis.

**Figure 12:**
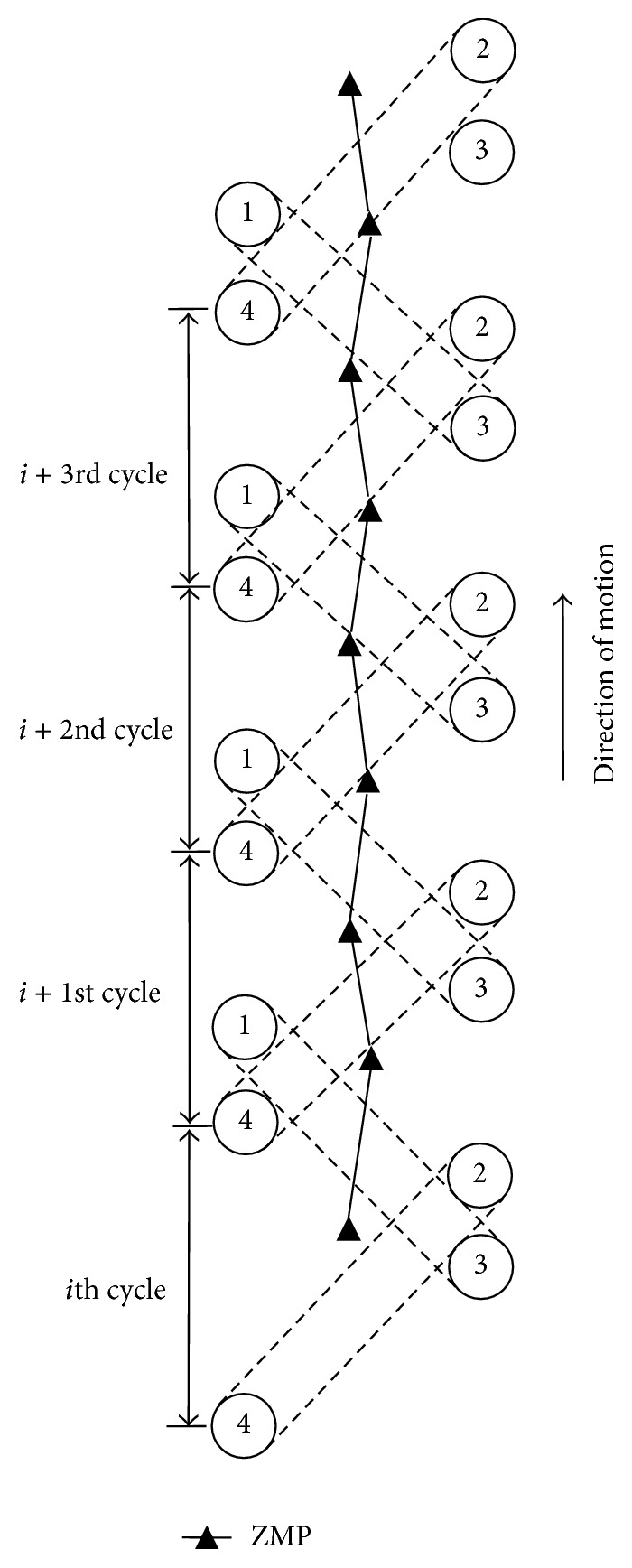
ZMP trajectory analysis diagram for trot gait.

In the case of trot gait, the ZMP trajectory analysis is shown in Figure 12.

ZMP is in the upper left of support diagonal when the left hind leg 2 and the right front leg 4 are the supporting legs. ZMP is in the upper right of support diagonal when the left front leg 1 and the hind front leg 3 are the supporting legs. So ZMP crosses support diagonal twice in one movement cycle. The change of ZMP in *Z* direction is in *S* curve, which shows pose of robot is adjusted in the right or the left continually.

## 6. Hybrid CPG-ZMP Control System

**Figure 13:**
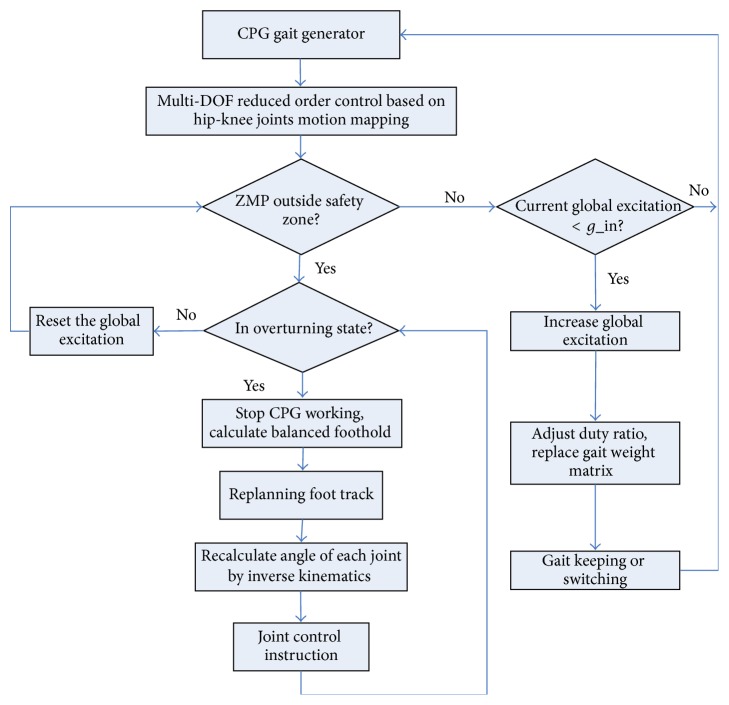
Flow chart of hybrid CPG-ZMP control algorithm.

The flow chart of hybrid CPG-ZMP control algorithm is shown in Figure 13. The motion track of each joint is generated by the improved CPG and motion mapping; meanwhile we specify the global threshold of CPG (*g*_in). Rhythmic motion of robot is realized by CPG gait generator, and CPG can receive feedback signal from body sensor while working.

ZMP can be calculated by datum from force sensor and gyroscope. The control system can tell whether the ZMP is outside of safe area. If true but still not up to the critical point of turnover, ZMP detector sends a signal to reduce the global threshold quickly for restraining roll, and then the neural signal activity in CPG is lowered. If not outside of safe area, the control system will detect whether the current global threshold is smaller than the preset value; if true, the system will increase the global threshold. If ZMP is outside of safe area and the robot is in the state of turnover, which is mainly made by external impact and disturbance, CPG stops working and the control system will recalculate balanced foothold. To recover the pose of robot, the angle of robot's each joint is recalculated by inverse kinematics. And CPG is back to work until robot pose is normal. The theory of ZMP not only can be used as the stability criterion of robot gait, but also can be used to plan the corresponding gait when robot is in the state of turnover.

## 7. Experiment Study

### 7.1. Simulation Experiment

**Figure 14:**
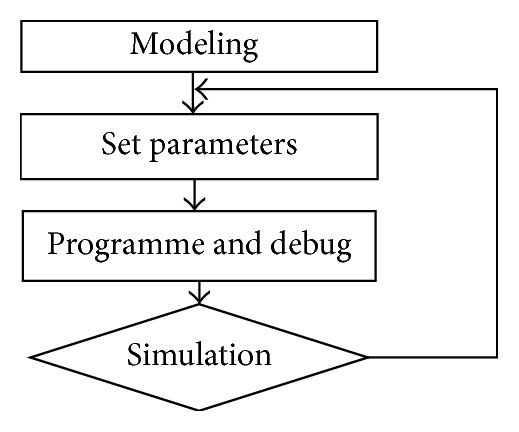
Webots simulation flow chart.

The flow chart of gait plan simulation based on Webots is shown in Figure 14. The key parameters of walk gait and trot gait are shown in Table 2 by cut-and-try method.

#### 7.1.1. Walk Gait

**Figure 15:**
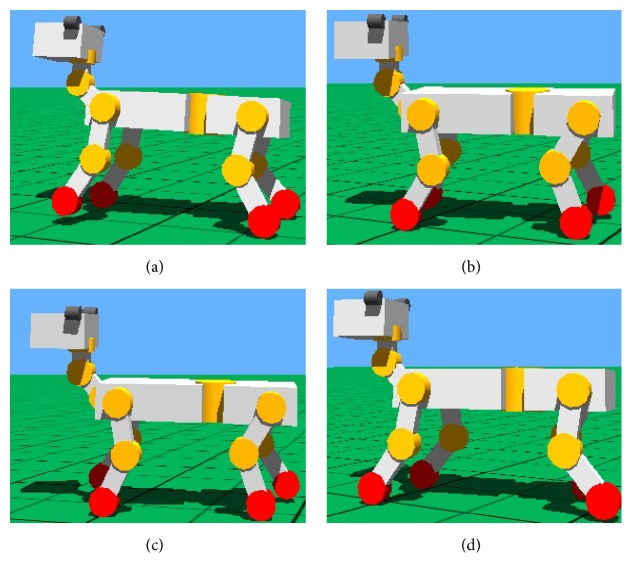
Movement orders of legs in walk gait. (a) Left foreleg in swing phase. (b) Right hind leg in swing phase. (c) Right foreleg in swing phase. (d) Left hind leg in swing phase.

The walk gait simulation of quadruped robot based on Webots with the improved CPG in the paper is shown in Figure 15. Quadruped robot walks in the 1-3-2-4 order (1: left foreleg, 2: right foreleg, 3: right hind leg, and 4: left hind leg) and there are three legs on the ground which are in stand phase at any time from the simulation chart. The simulation of walk shows that robot moves at a constant velocity and the pose is steady, so the algorithm has good practicability.

#### 7.1.2. Trot Gait

**Figure 16:**
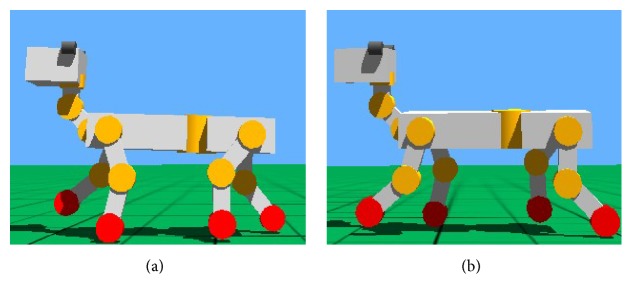
Movement orders of diagonal legs in gait trot. (a) RF and LH in swing phase. (b) LF and RH in swing phase.

The trot gait simulation of quadruped robot based on Webots with the improved CPG in the paper is shown in Figure 16. The left foreleg and the right hind leg are in support phase when the right front leg and the left hind leg are in swing phase. The simulation shows that the speed of robot in trot is obviously higher than the speed in walk, but the stability of body is lower.

#### 7.1.3. Typical Gaits Transition

**Figure 17:**
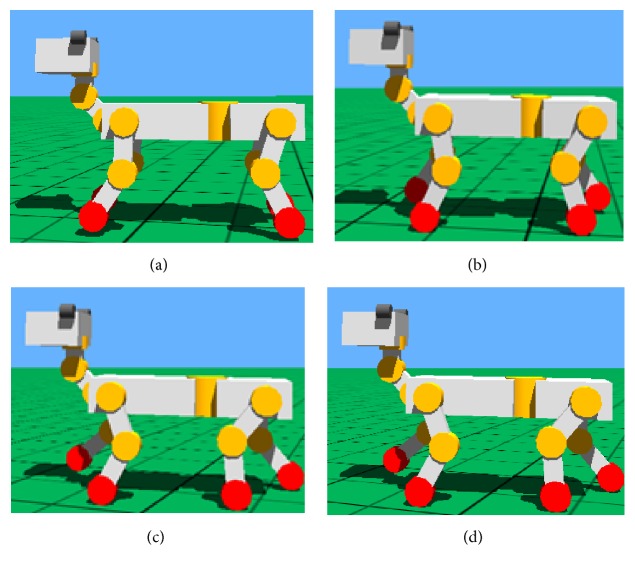
Gaits transition from walk to trot. (a) Walk. (b) Transient state 1. (c) Transient state 2. (d) Trot.

Gaits transition from walk to trot is shown in Figure 17 (*v* = 0.24 ms^−1^). The adjusting time is about 0.5 T. Gaits transition is rapid and smooth.

#### 7.1.4. Stability Simulation of CPG Control

**Figure 18:**
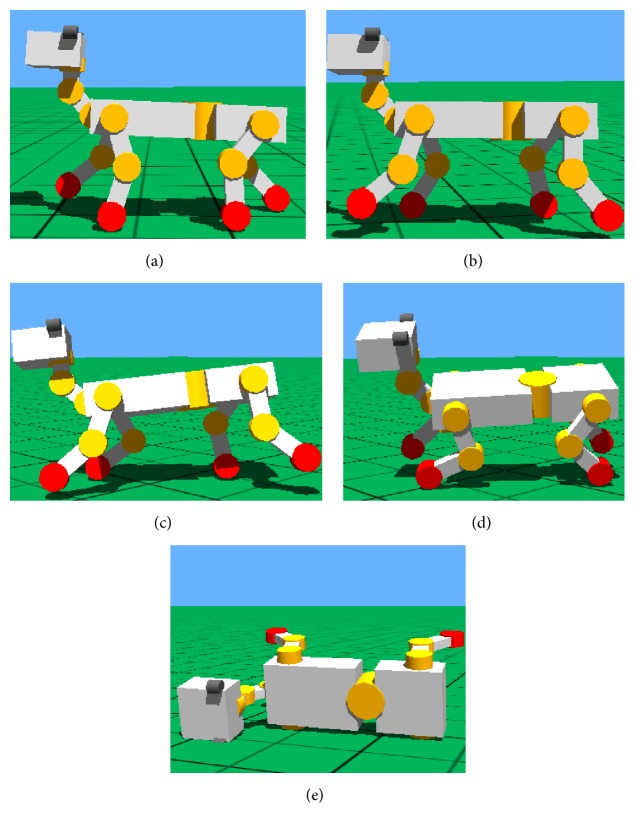
Simulation of trot gait controlled only by CPG. (a) *v* = 0.24 ms^−1^. (b) *v* = 0.288 ms^−1^. (c) *v* = 0.312 ms^−1^. (d) Severe instability (*v* = 0.312 ms^−1^). (e) Rollover (*v* = 0.312 ms^−1^).

**Figure 19:**
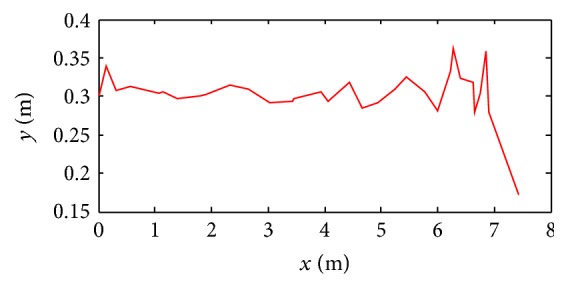
Height change of robot centroid in *y*-axis with displacement in *x*-axis.

The simulation result is shown in Figure 18. The stability of robot decreases when the speed is higher. When the speed is up to 1.3*v* (*v* = 0.24 ms^−1^), gaits become disordered because robot's inertia and the impact force of toes increase. And, then, the body jolts violently in the direction of move. Finally, the robot is in rollover after about one motion cycle. The height change of robot centroid in the direction of *y*-axis during the movements is shown in Figure 19.

#### 7.1.5. Stability Simulation of CPG-ZMP Control

**Figure 20:**
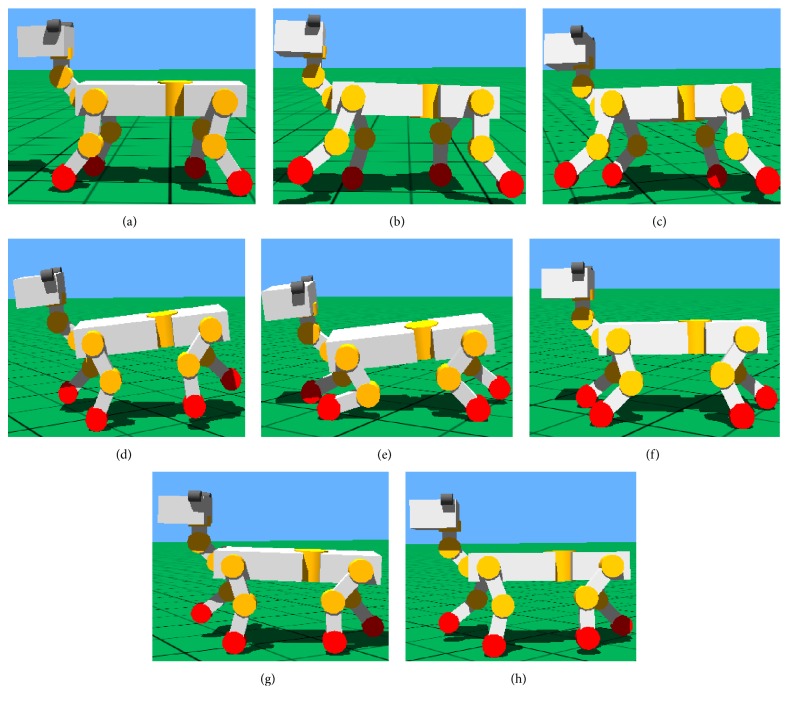
Trot simulation controlled by CPG-ZMP. (a) *v* = 0.24 ms^−1^. (b) *v* = 0.288 ms^−1^. (c) *v* = 0.312 ms^−1^. (d) Severe instability (*v* = 0.312 ms^−1^). (e) Stop CPG and replan gait. (f) Adjusting joint angle. (g) Restart CPG working. (h) Recover normal gait.

**Figure 21:**
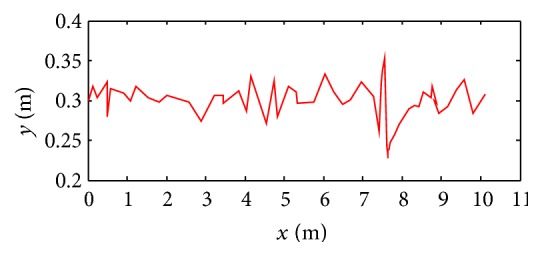
Height change of robot centroid in *y*-axis with displacement in *x*-axis.

The simulation results are shown in Figure 20. The robot's stability decreases when the speed is higher. But the robot adjusts gait adaptively by CPG-ZMP control and is kept from falling over and the rollover effectively. The height change of robot centroid in the direction of *y*-axis during the movements is shown in Figure 21.

When the speed is up to 0.312 ms^−1^ (1.3*v*), the robot jolts violently in the direction of move, and then the robot becomes unstable gradually. CPG stops working and gaits of robot are replanned by inverse kinematics when ZMP of robot is out of the safe range and is turning over. After about one and a half motion cycles, the CPG restarts working when ZMP is in safe range; then the robot's gait becomes normal.

### 7.2. Real Experiment

**Figure 22:**
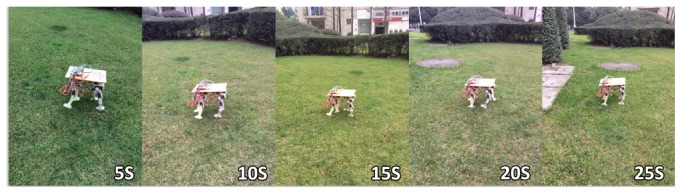
The overview of the motion in a rough terrain.

For testing our proposed controller, we designed a robot, which has 16 DOFs, and each leg has 3 actuated rotary joints. Each rotary joint is controlled by a steering engine. In addition, an IMU sensor is attached to the robot's body to measure the orientation (roll-pitch-yaw angles), body linear acceleration, and rotational velocity. Furthermore, each leg is equipped with a load cell. To evaluate the efficiency of the proposed controller, we performed some tests under rough terrain using a walk gait. The desired velocity is set to 0.08 ms^−1^. The robot can adaptively adjust velocity from 0.03 ms^−1^ to 0.08 ms^−1^ according to movement environment and its posture. Experiment results show the CPG-ZMP controller adapts to the environment change very well. The overview is shown in Figure 22.

## 8. Conclusions

We conclude the following:

1. The improved CPG system based on Wilson-Cowan model shortens the oscillation time and makes the system respond quickly and enhances the real time; meanwhile the gait switch is more smooth and rapid.
2. The intercoupling mapping relations between the hip joint and the knee joint are built by the improved multi-DOF reduced order control theory, which improves the efficiency of control and the real time. A quadruped robot takes 8 DOFs to realize rhythmic movements, so if 8 DOFs are controlled by CPG, CPG nets are too complex to influence the real-time performance of system.
3. The robot adjusts gait adaptively and the stability of robot's movement is improved greatly by CPG-ZMP control.

## Figures and Tables

**Table 1:** Parameters of the CPG differential equations.

*T*_*u*_, *T*_*v*_	*a*	*d*	*b*	*c*	*S*_*u*_, *S*_*v*_	*μ*	*p*	*m*	*g*_*k*_
0.2	5.6	−2.4	5.6	2.4	0.02	1	0.5	1	0.1

**Table 2:** Parameters of the two kinds of gaits.

	*A*_*h*_	*A*_*k*_	*α*_*h*_	*α*_*k*_	*p*
Walk	15.50°	11.12°	1.27	1.75	0.25
Trot	45.00°	1.90°	1.00	2.11	0.50
